# Multiphoton Laser Microscopy and Fluorescence Lifetime Imaging for the Evaluation of the Skin

**DOI:** 10.1155/2012/810749

**Published:** 2011-11-28

**Authors:** Stefania Seidenari, Federica Arginelli, Sara Bassoli, Jennifer Cautela, Paul M. W. French, Mario Guanti, Davide Guardoli, Karsten König, Clifford Talbot, Chris Dunsby

**Affiliations:** ^1^Department of Dermatology, University of Modena and Reggio Emilia, 41124 Modena, Italy; ^2^Department of Physics, Imperial College London, South Kensington Campus, London SW7 2AZ, UK; ^3^Department of Biophotonics and Lasertechnology, Saarland University, Campus A5.1, 66123 Saarbrücken, Germany; ^4^JenLab GmbH, Schillerstraße 1, 0745 Jena, Germany

## Abstract

Multiphoton laser microscopy is a new, non-invasive technique providing access to the skin at a cellular and subcellular level, which is based both on autofluorescence and fluorescence lifetime imaging. Whereas the former considers fluorescence intensity emitted by epidermal and dermal fluorophores and by the extra-cellular matrix, fluorescence lifetime imaging (FLIM), is generated by the fluorescence decay rate. This innovative technique can be applied to the study of living skin, cell cultures and *ex vivo* samples. Although still limited to the clinical research field, the development of multiphoton laser microscopy is thought to become suitable for a practical application in the next few years: in this paper, we performed an accurate review of the studies published so far, considering the possible fields of application of this imaging method and providing high quality images acquired in the Department of Dermatology of the University of Modena.

## 1. Introduction

Scientific research keeps on developing new technologies to enable a high resolution optical diagnosis based on *in vivo* imaging of the skin and its components, aiming both at avoiding scars due to unnecessary biopsies and skin resections and providing a support for histopathology, that, in spite of remaining the Gold Standard for diagnosis, does not always show a satisfactory interobserver agreement. One of the most recent clinical imaging technologies is multiphoton tomography (MPT), which is becoming established as the preferred method for image living cells with submicron resolution [1–10]. 

## 2. Principles of Functioning of Multiphoton Laser Tomography

Multiphoton laser microscopy (multiphoton laser tomography, MPT) provides instant imaging of living skin at a cellular and subcellular level. MPT can exploit autofluorescence of intrinsic tissue fluorophores and nonlinear harmonic generation from tissue matrix components such as collagen, thereby enabling functional and structural imaging of unstained biological tissue [1–10]. Whereas, for conventional confocal fluorescence microscopy, fluorophores are excited by absorption of individual photons in the visible or ultraviolet spectrum, MPT excitation entails the simultaneous absorption of two or more photons of longer wavelength, usually in the near-infrared spectrum. This longer wavelength infrared radiation undergoes less scattering than visible light and can thus facilitate high-resolution imaging deeper into biological tissue. Efficient MPT excitation usually requires ultrashort femtosecond laser pulses, and these are also efficient in producing the nonlinear effect of second harmonic generation (SHG), which can be observed in periodic structures such as collagen [4, 9]. The combination of autofluorescence imaging and SHG in MPT can provide morphology and structure of both cells and extracellular matrix of the skin [1–10]. 

Autofluorescence imaging is based on the excitation of some endogenous cellular or extracellular components that exhibit fluorescence. Fluorophores are integral components of the molecules to which they confer the characteristic autofluorescence. After energy absorption by the fluorophores, they can then emit energy in turn, generating a visible signal. Energy emission from the fluorophores happens at defined wavelengths, different from those of absorption. The quantity and the wavelength of the emitted energy depend on the chemical characteristics of the fluorophore, on its environment, and particularly on the type of the surrounding molecules [9]. 

The endogenous fluorescent biomolecules present in human skin include NADH (reduced nicotinamide adenine dinucleotide), NADPH (reduced nicotinamide adenine dinucleotide phosphate), collagen, keratin, melanin, elastin, flavines, porphyrin, tryptophan, cholecalciferol, and lipofuscin.

These fluorophores generally require excitation wavelengths in the UV spectral range, which is highly energetic and damaging to the skin. Hence, when used within established limits, excitation with NIR light is less harmful for biological tissue and it represents a clear advantage if used as an *in vivo* diagnostic method [1–10]. 

Using the fluorescence emitted from endogenous molecules through fluorophores makes the use of any other contrast agent or exogenous marker unnecessary, simplifying both examination and patient preparation. 

Besides autofluorescence, emission in the visible range also comprises SHG signals. The SHG signal comes from noncentrosymmetric molecules such as collagen and myosin and is characterized by an emission wavelength corresponding to half of that of the incident photon; this particular signal allows the visualization of dermal collagen bundles and their distinction from cellular components and elastin fibers [11].

With MPT, bidimensional images are acquired and correspond to optical sectioning parallel to the tissue surface (reported to a defined *xy*-plane). Pictures obtained at various depths, called *z*-stacks, can be acquired by sequentially modifying the depth of the focal plane in the tissue reaching levels of 200 *μ*m measured from the departure point at the skin surface [3, 5, 9]. 

**Figure 1:**
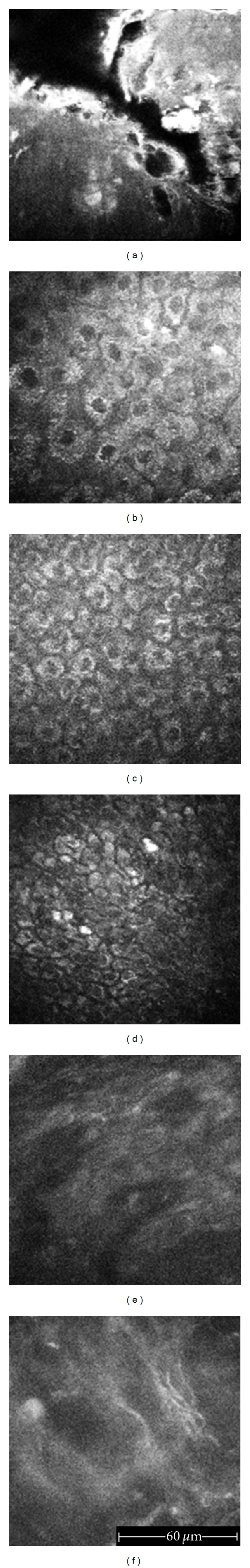
*In vivo *healthy skin. Images of the epidermis are acquired by means of the multiphoton laser microscope *DermaInspect *(JenLab GmbH, Jena, Germany) at a 760 nm excitation wavelength. (a) Stratum corneum, 0 *μ*m depth: strong autofluorescence coming from keratin and corneocytes, hexagonal-shaped flat cells. (b) Stratum granulosum, 20 *μ*m depth: large keratinocytes showing strong cytoplasmatic autofluorescence due to the presence of keratohyalin granules, NADPH, and keratin; the nuclei appear dark because they contain less fluorophores than the cytoplasm. (c) Stratum spinosum, 30 *μ*m depth: smaller keratinocytes and increased cellular density. (d) Stratum basale, 40 *μ*m depth: the keratinocytes are small, polygonal-shaped, and with dark nuclei. (e) Dermal papillae, 55 *μ*m depth: they are visible beyond the dermal-epidermal junction as oval-shaped, dark areas with a fluorescent contour, corresponding to a transversal section of the *rete ridges* surrounded by fluorescent basal cells. (f) Dermis, 85 *μ*m depth, 800 nm excitation wavelength: bright autofluorescence coming from fibrous structures corresponding to collagen and elastin.

Grey scale images are generated, reproducing the fluorescence intensity in different tissue components (Figure 1). The estimated resolution is 0.5 *μ*m in lateral direction and 1-2 *μ*m in the axial direction [3, 5, 9]. 

## 3. Excitation Wavelength

**Figure 2:**
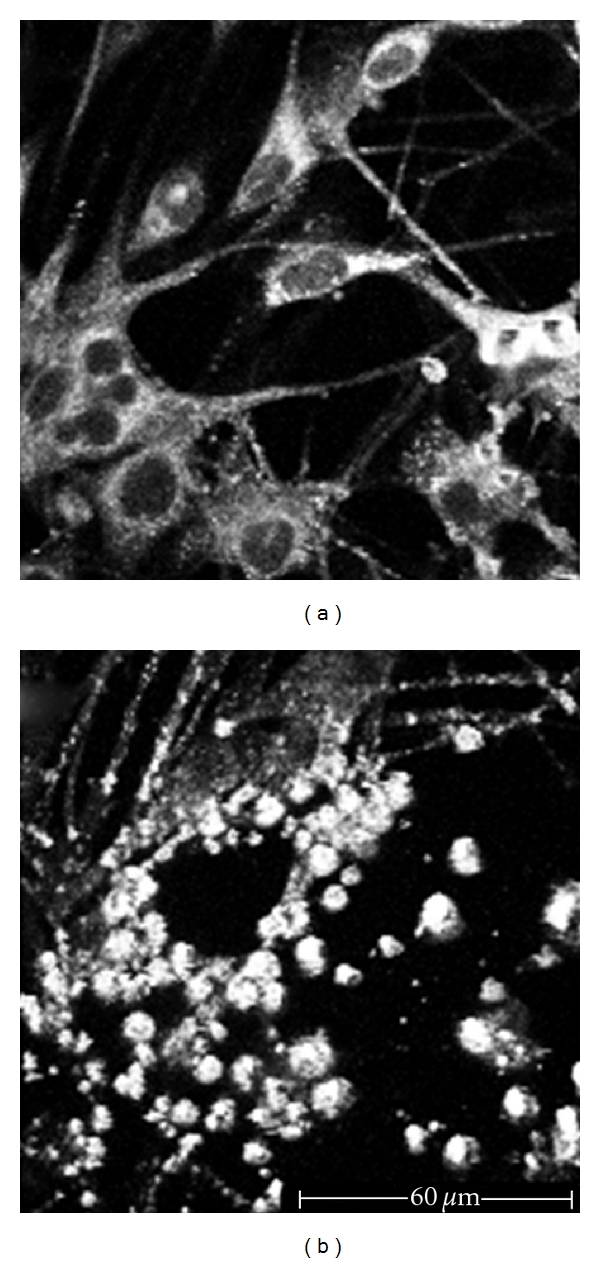
Melanocytes culture. (a) 760 nm excitation wavelength: melanocytes appear as dendritic cells, with a clearly distinguishable cellular body (dark nucleus and fluorescent cytoplasm) and peripheral ramifications. (b) 820 nm excitation wavelength: increasing the excitation wavelength, the cellular body and the dendrites disappear. We can only notice the strong autofluorescence due to melanin granules inside the dendrites.

The epidermal structures can be excited efficiently at a wavelength of 760 nm [1–10]. When the excitation wavelength is increased above 800 nm, most keratinocytes lose their excitability and progressively become invisible. Since melanin, compared with other organic fluorophores, has an absorption spectrum that decreases from the UV region to NIR, its selective excitation wavelength is 800 nm [12, 13] (Figure 2). Thus, at 800 nm, we can note the presence of single cells in the basal layer showing intense fluorescence, identified as melanocytes. This excitation wavelength can be used to study benign and malignant melanocytic lesions.

Collagen and elastin are the main dermal components, and their excitation takes place at a variable wavelength from 760 to 840 nm. A 800 nm wavelength is generally employed to adequately visualize the fibrous structures of the dermis. At this wavelength, collagen fibers that generate the SHG signal are selectively excited, whereas, at 760 nm, dermal autofluorescent components such as elastin are enhanced in the image. 

## 4. Fluorescence Lifetime Imaging

**Figure 3:**
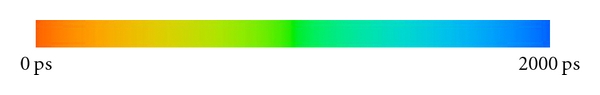
A false-colour scale, in this case corresponding to 0–2000 ps, is used to define the fluorescence lifetime decay rate of each fluorophore: for each colour, there is a corresponding value of lifetime decay.

Another possible imaging modality which can be obtained with MPT is based on the study of the kinetics of the fluorescence time decay in various tissue components. Fluorescence lifetime imaging (FLIM) engenders the generation of new images that supply us with information about different states of tissue characterized by different fluorescence decay rates. Since FLIM is immune to intensity artefacts, it enables a more robust numerical description of the images than intensity imaging [14, 15]. Our FLIM system, developed jointly by the Photonics Group of the Imperial College of London and JenLab GmbH, has been incorporated into the commercially available *DermaInspect*. False colour coding (Figure 3), where each colour corresponds to a certain fluorescent lifetime at each image pixel, permits the immediate visual identification of cellular, subcellular, or extracellular structures according to their colour in the FLIM image. The distribution of fluorescence lifetimes within an image is visualized through a histogram that plots the fluorescence lifetime (*x* axis) against the number of corresponding pixels for which that lifetime occurs (*y* axis). The time-resolved analysis of the fluorescence signal generates four dimensional data sets, where the tissue is not only studied according to its structure in the *x*-, *y*-, or *z*-axis, but also according to the fluorescence dynamics of its components which can, for example, report on metabolic characteristics.

**Figure 4:**
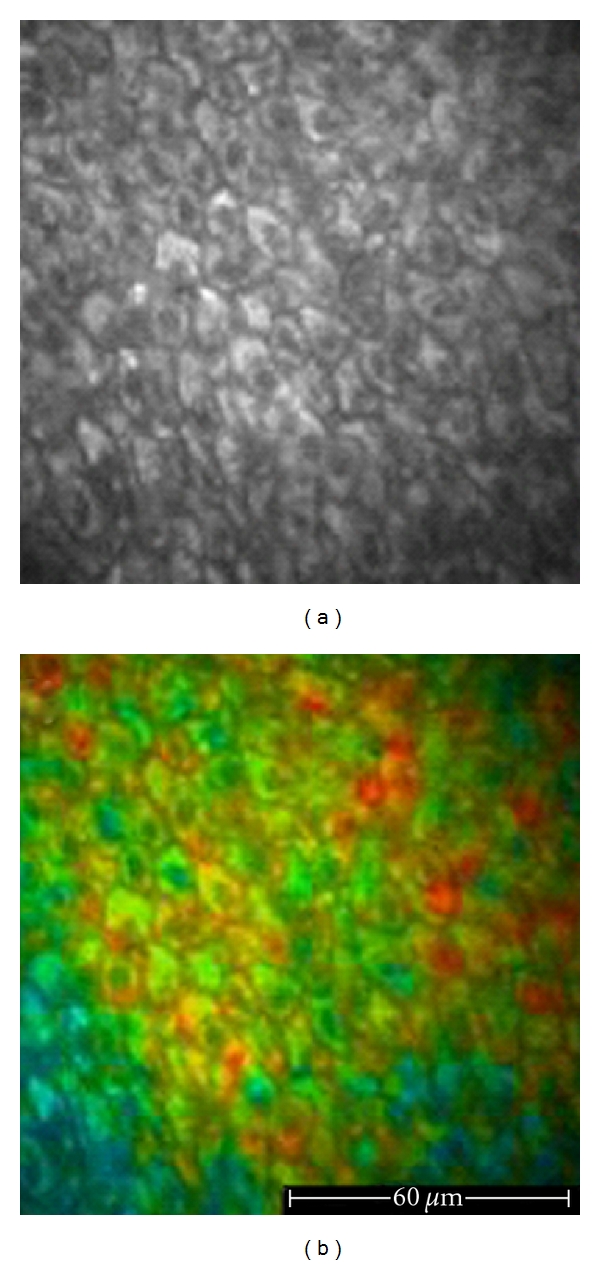
*In vivo *healthy skin, 760 nm excitation wavelength. The acquisition of the images has been achieved by means of the *DermaInspect *equipped with the FLIM system, planned, and built by the Photonics Group of the Imperial College of London. (a) The autofluorescent signals of keratinocytes, melanin containing keratinocytes, and melanocytes appear monomorphous, and, in the grey-scale images, they cannot be distinguished from each other. (b) The FLIM image enables the identification of melanin-containing cells based on the different fluorescence decay time values (keratinocytes appear green-blue and melanin containing keratinocytes and melanocytes appear yellow-red).

**Figure 5:**
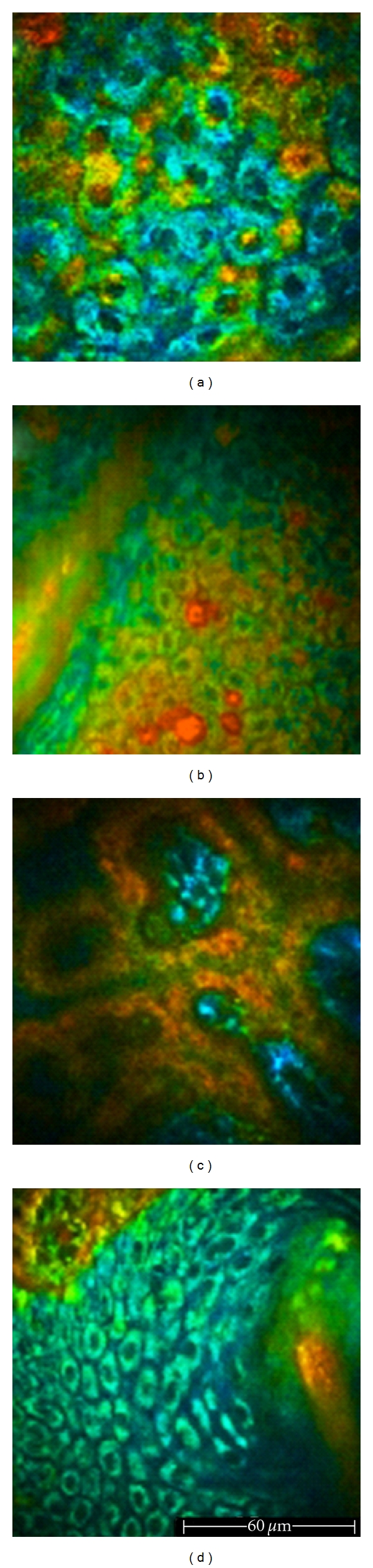
FLIM images of healthy skin acquired *in vivo *by the Imperial College FLIM system incorporated in the *DermaInspect*. (a) 760 nm excitation wavelength. In the false-colour images, keratinocytes appear green-blue, showing a longer fluorescence lifetime with respect to melanin aggregates corresponding to yellow-red areas with a shorter lifetime value. (b) 760 nm excitation wavelength. Keratinocytes of the basal layer appearing yellow-red because of the presence of pigmentation. (c) 760 nm excitation wavelength. Dermal papillae surrounded by pigmented keratinocytes and melanocytes with low FLIM values. (d) 760 nm excitation wavelength. A hair-follicle with bluish perifollicular cells showing a shorter lifetime.

**Figure 6:**
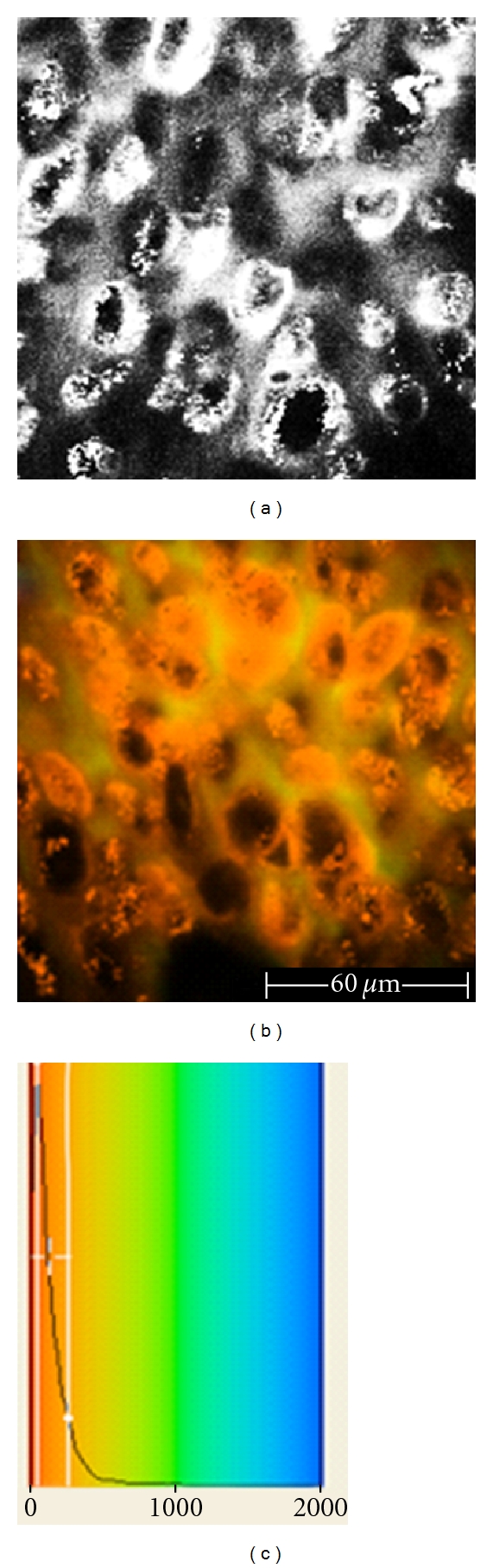
Malignant melanoma examined *ex vivo*, 40 *μ*m depth, 760 nm excitation wavelength. (a) Grey scale image acquired by the *DermaInspect. *We observe an irregular distribution of pleomorphic cells with different shape and size. (b) FLIM image acquired by the *DermaInspect *equipped with the Imperial College FLIM system. Melanoma cells contain melanin and appear yellow (short lifetime values). (c) The diagram shows the fluorescence lifetime on the *x* axis (from 0 to 2000 ps) and the number of pixels corresponding to the fluorescence lifetime on the *y* axis.

With FLIM, keratinocytes exhibit long-medium values, visually corresponding to the blue-green range, whereas melanocytes have a medium to short fluorescence decay time coded in the yellow-red range (Figures 4 and 5) [16]. Melanoma cells, selectively excited at 800 nm, are highly fluorescent and show atypical aspects and pleomorphism (Figure 6).

## 5. Application Fields

**Figure 7:**
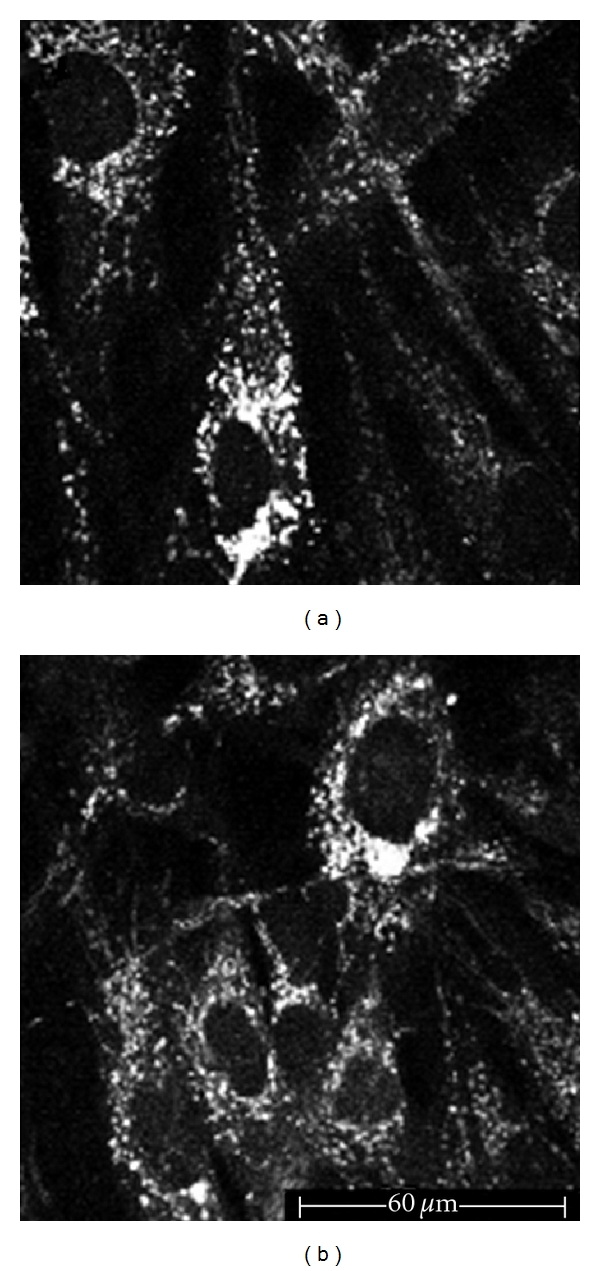
Fibroblast culture, 760 nm excitation wavelength. (a) Fibroblasts, before UVB irradiation, appearing as elongated cells; both the cellular bodies and the dendrites show an autofluorescent signal coming from the cytoplasmatic granules. (b) After UVB irradiation, some fibroblasts undergo morphologic alterations corresponding to necrosis.

The MPT/FLIM technique has numerous applications in dermatology, being suitable for the study of physiological and pathological conditions of the skin on *ex vivo *and* in vivo* samples. Cell cultures can also be studied [17, 18]. The effects on metabolic activity, morphology, and oxidative stress of mesenchimal stem cells undergoing various differentiation stimuli can be monitored in a noninvasive manner [17]. Fibroblast cultures are widely used as an experimental model in various types of investigations, to study the expression of specific genes or the effect of drugs, especially of new compounds with potential chemotherapeutic activity, and to check the mutagenicity and carcinogenicity of different substances. Using MPT and FLIM, a precise and rapid assessment of the morphologic and metabolic changes which undergo fibroblasts after exposure to various environmental factors can be achieved without the need of cell processing and staining [18] (Figure 7). 

 When trying to characterize diseased states of the skin, knowledge about normal morphology is of utmost importance. With regards to healthy epidermis, it has been shown that cell and nucleus diameters, cell density, and FLIM characteristics vary not only according to epidermal cell depth, but also depending on skin site. In elderly subjects, epidermal cells show morphologic alterations in shape and size and appear more sparse; moreover, both cell and nucleus diameters are smaller at the upper layers. Finally, the number of basal cells is decreased, whereas FLIM values at both the upper and lower layers increase during the ageing process [16]. These data can be used for the comparison with MPT/FLIM aspects of epidermal cells in pathological conditions. 

**Figure 8:**
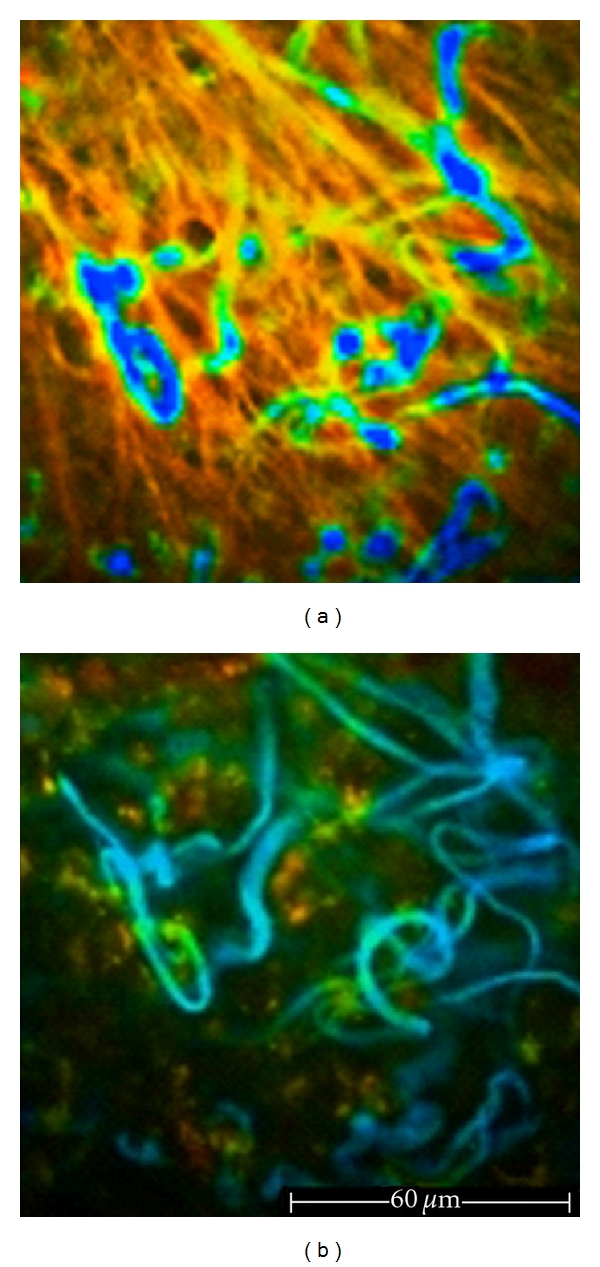
Dermis. (a) 100 *μ*m depth, 760 nm excitation wavelength. Thick and straight collagen fibers show low FLIM values (orange), whereas elastic fibers have high FLIM values and are represented in blue. (b) 100 *μ*m depths, 760 nm excitation wavelength (same spot). Employing the filter excluding SHG signals, curled blue elastic fibers are highlighted and their fine network is visible.

Going deeper into the dermis, the morphology of collagen and elastic fibers can be observed (Figure 8). Morphological changes in the dermal collagen and elastin fibre network are characteristic for skin ageing and for pathological skin conditions of the dermis. During the ageing process, fibre tension and morphology, network pattern, and clot formation, as assessed by MPT, change [19]. Employing *in vivo* autofluorescence and SHG imaging, a relationship between morphological characteristics of human dermis assessed by MLT and age was demonstrated [20]. 

Investigating facial skin specimens from patients of different ages by MPT imaging, Lin et al. found age-dependent changes in the dermal extracellular matrix [21]. In the case of 20-year-old individuals, AF and SHG signals were interspersed in the papillary dermis, whereas, in 70-year-old individuals, SHG signals could only be detected in a very thin zone beneath the basement membrane, and large amounts of fluorescent elastic fibers, corresponding to solar elastosis, were found in the dermis. The trend of decreasing SHG signals and increasing autofluorescence signals was well correlated with the histological findings of the decrease of collagen fibers and the increase of elastic fibers with increasing age. 

**Figure 9:**
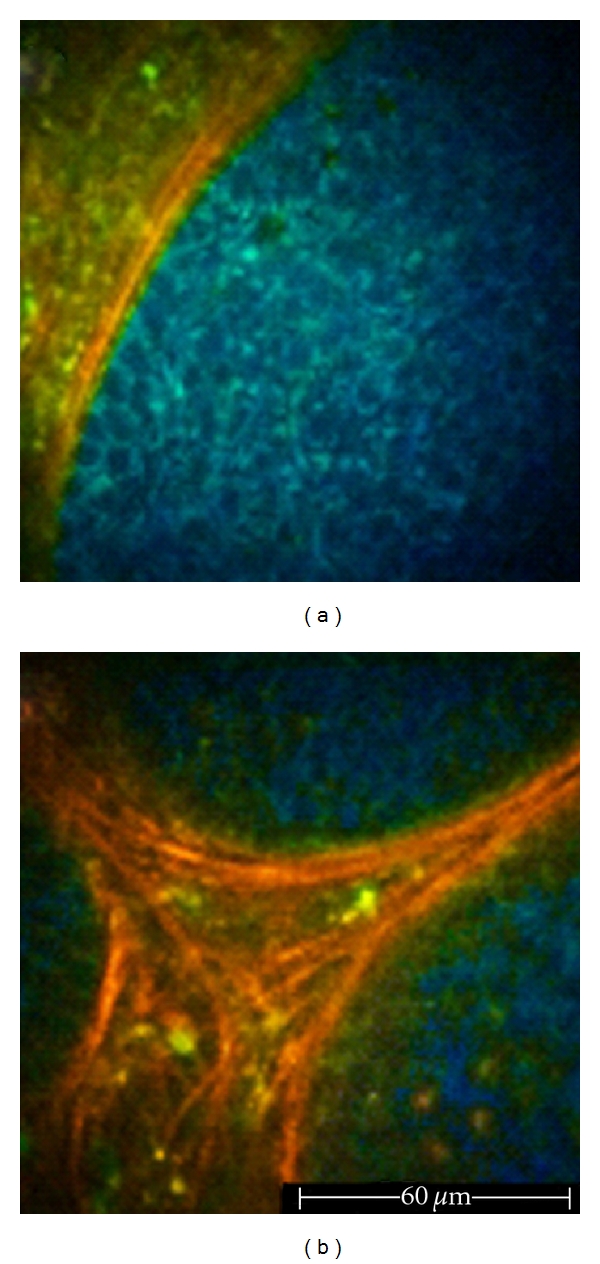
Basal cell carcinoma examined *ex vivo*. (a) 760 nm excitation wavelength. Nest of basaloid cells (blue) surrounded by a network of collagen fibers (red). (b) 800 nm excitation wavelength. Thick bundles of collagen fibers surrounding three basaloid nests.

The application of MPT/FLIM to the field of skin tumors appears promising [15, 22–26] (Figure 9). Cicchi et al. performed a study using multidimensional nonlinear laser imaging approach to visualize *ex-vivo *samples of basal cell carcinoma (BCC) [22]. In order to detect morphological, biochemical, and physiochemical differences between healthy skin and BCC, they used a combination of several nonlinear laser imaging techniques involving fluorescence lifetime (FLIM), multispectral two-photon and second-harmonic generation imaging to image different skin layers. Their study showed that, in comparison with normal skin, BCC showed a blue-shifted fluorescence emission, a higher fluorescence response at 800 nm excitation wavelength, and a slightly longer mean fluorescence lifetime. In the same year, Skala et al. carried out a study about structural and functional characteristics of precancerous epithelia [23]. First, they demonstrated a significant decrease in the contribution and lifetime of protein-bound NADH in both low- and high-grade epithelial precancers compared with normal epithelial tissues; second, significantly increased intracellular variability in the redox ratio and NADH fluorescence lifetimes were observed in precancerous cells compared with normal cells. 

 In 2008, the group of the Imperial College of London published another *ex vivo* study regarding BCC [15]. They demonstrated that BCCs exhibit shorter fluorescence lifetimes than the surrounding noninvolved skin, allowing the two areas to be distinguished by wide-field imaging on the basis of lifetime information. Dimitrow et al. investigated some procedures of selective melanin imaging and spectral fluorescence lifetime imaging in combination with high-resolution multiphoton laser tomography by analysing 46 melanocytic lesions of human skin [13]. Remarkable differences in lifetime behaviour of keratinocytes in contrast to melanocytes were observed. The fluorescence lifetime distribution was found in correlation with the intracellular amount of melanin. Examining the unique light absorption behaviour of melanin, they found a selective fluorescence of melanin containing cells at an excitation wavelength of 800 nm. Their results revealed that keratinocyte lifetime values correspond to NAD(P)H and melanocyte lifetime values to the endogenous fluorophore melanin. Thus, whereas FLIM on single-data points allowed a clear separation of different melanized cell types (e.g., keratinocytes, melanocytes), it seemed to be unsuitable for a classification of benign versus malign melanocytic skin lesions. Both melanocytic nevi and MM were characterized by enhanced cell-density of melanocytes primarily in the basal epidermal layer. Being the alteration of the upper epidermal layers, a unique characteristic of malignant proliferation, the crucial point of differentiation was identified in the presence of ascending melanocytes. Spectral analysis of MM revealed a main fluorescence peak around 470 nm in combination with an additional peak close to 550 nm throughout all epidermal layers. From this, they concluded that morphological alterations, in combination with a selective excitability at 800 nm, a short medium lifetime, and a distinct peak around 550 nm in suprabasal epidermal layers, are highly suggestive of MM.

## 6. Advantages, Disadvantages, and Future Objectives of MPT/FLIM

The results obtained with MPT are extremely coherent with those of histopathologic analysis, which suggests that this new technique, once fully developed, may replace histology [9].

Autofluorescence images are immediately visualized during the examination process in real time. FLIM images require a further elaboration to produce a pseudocolor image and to allow the operational system to develop a diagram of the fluorescence decay time [15, 16]. Images generated by MPT/FLIM can be analyzed either on a morphological basis, or on a functional one, studying the kinetics of the fluorescence decline and the differences produced by the excitation of a sample with various wavelengths [14, 15]. Considering the technique as noninvasive and the laser illumination harmless, *in vivo* examination can be repeated on the same explored skin site within short or long intervals allowing for the long-term studies of cutaneous affections [9]. Thanks to the opportunity to obtain horizontal and vertical optical sections, it is possible to study the tissue sample in three-dimensions with a subcellular spatial resolution. Moreover, the nonlinear excitation produced by the NIR radiation allows a deeper penetration than the one of confocal microscopy, with a better discrimination of the various tissue components and higher visualization of the deep dermis [9].

On the other hand, the MPT/FLIM technique presents some problems relative to its use. Time of acquisition of the images is longer compared to that of traditional fluorescent microscopy and confocal microscopy, and this implies that the patient is forced to keep still and motionless in order to obtain stable images for the entire duration of the examination. Thus, one goal is to reduce the acquisition time to avoid imaging deformities caused by voluntary and involuntary movements due to cardiac pulsations or muscular thrills. Another important aim is to make the instrument more manageable by introducing a mechanical arm or other specific devices to explore difficult body sites or irregular surfaces [9]. Finally, since at present it is possible to investigate only a small area of a lesion, which may result in a poor diagnosis, it is necessary to have the possibility to acquire contiguous images and assemble them automatically through a specific software, to obtain a surface large enough to be representative of the whole lesion.
